# Water and Sanitation Hygiene Practices for Under-Five Children among Households of Sugali Tribe of Chittoor District, Andhra Pradesh, India

**DOI:** 10.1155/2017/7517414

**Published:** 2017-05-31

**Authors:** Venkatashiva Reddy B, Yadlapalli S. Kusuma, Chandrakant S. Pandav, Anil Kumar Goswami, Anand Krishnan

**Affiliations:** ^1^Department of Community Medicine, Veer Chandra Singh Garhwali Government Medical Sciences and Research Institute, Uttarakhand, India; ^2^Centre for Community Medicine, All India Institute of Medical Sciences, New Delhi, India

## Abstract

**Background:**

Increased mortality is associated with poor household water, sanitation, and hygiene (WaSH) practices. The objective was to study the WaSH practices for under-five children among households of Sugali Tribe, Chittoor district, Andhra Pradesh, India.

**Methods:**

A community-based cross-sectional study was conducted in four mandals in 2012. A total of 500 households with under-five children were identified. Data was collected from mothers/caregivers. A summary WaSH score was generated from four specific indices, water, sanitation, hygiene, and hand washing practices, and determinants were identified.

**Results:**

Of the total households, 69% reported doing nothing at home to make the water safe for drinking. Over 90% of the households reported storing water in a utensil covered with a lid and retrieving water by dipping glass in the vessels. Open defecation was a commonly reported practice (84.8%). About three-fifths of the study's households reported using water and soap for cleaning dirty hands and one-third (37.4%) reported using water and soap after defecation. The median WaSH score was 15. In the hierarchical stepwise multiple linear regression, only socioeconomic variables were significantly associated with WaSH score.

**Conclusion:**

WaSH related practices were generally poor in people of the Sugali Tribe in Andhra Pradesh, India.

## 1. Introduction

The tribal population groups of India are indigenous people of the land. Tribals are often referred to as adivasi, vanyajati, vanvasi, pahari, adimjati, and anusuchit jan jati, the latter being the constitutional term [1]. Tribal groups, as is also true for other population groups, are at different stages of social, economic, and educational development. While some tribal communities have integrated and adopted a mainstream way of life, at the other end of the spectrum, tribes are characterized by a preagriculture level of technology, a stagnant or declining population, extremely low literacy, and a subsistence level of economy [2]. The Sugali Tribe of Andhra Pradesh represents one such tribe whose members were originally nomads but have now settled into some sort of a permanent settlement. In Andhra Pradesh, Sugalis are numerically the largest Scheduled Tribe (ST) constituting 41.4 percent of the state's ST population [3].

Many Indian tribes lived in the remote hilly forest areas and remained isolated, untouched by civilization. As a result, they were largely unaffected by the developmental processes going on in the rest of the state. Therefore, these groups remained backward, particularly in health, education, and socioeconomic aspects [4]. However, over a period, many of these tribes have gradually integrated into the rest of the society. This process of integration presents another set of challenges. This integration could result in some acculturation with a mix of traditional beliefs with modern beliefs resulting in changes in practices and customs. Children would be affected most by these changes, not only because they are a vulnerable group, but also because many of the beliefs which undergo a change are related to childbirth and child rearing [5].

Early childhood is the most important phase for overall development throughout the lifespan. The infant mortality rate and under-five child mortality rate for STs in Andhra Pradesh as per NFHS III estimates were 94.1 and 112, respectively [5]. More than half of these early child deaths were due to conditions that could be prevented or treated with access to simple, affordable interventions. Leading causes of death in the world in under-five children are pneumonia, diarrhea, and malnutrition. About one-third of all deaths of children are linked to malnutrition [6]. A significant proportion of deaths can be prevented through safe drinking water, adequate sanitation, hygiene, immunization, proper infant feeding, and enabling environments [7]. Therefore, interventions in the first five years of life can have a significant impact on the prevention of childhood morbidity and mortality [8].

The water, sanitation, and hygiene (WaSH) practices in India are documented [9–11]; there is still a paucity of studies from the tribal population. The objective of the current study was to study the water, sanitation, and hygiene practices for under-five children among households of the Sugali Tribe in Chittoor district, Andhra Pradesh, India, and identify WaSH practices' determinants.

## 2. Materials and Methods

This was a cross-sectional community-based study conducted in Madanapalle revenue division of Chittoor district in Andhra Pradesh from 2012 to 2014. Four mandals (administrative units in a district) with a high proportion of the Sugali population were selected. All thandas (Sugali settlements are known as thandas and are usually located at one end of the village) and urban settlements inhabited by the Sugalis were included. Most of the urban settlements had a single ethnicity in nature. The sample size was calculated considering the point prevalence of ARI among under-five children as 12.4% [12] assuming an alpha error of 0.05 and relative precision of 25%; the required sample size would be 470. Since the population of this tribe was small, all households were visited. As we wanted to study water, sanitation, and hygiene practices with respect to under-five children, households with at least one under-five child were included in the study. The youngest under-five child was studied if more than one was present.

A house-to-house survey was conducted in each of the selected thandas. A semistructured pretested interview schedule was used to collect information from the mother. In some cases (if the mother was not available or in case of orphaned children), information was collected from the caregiver. The questionnaire was pretested before starting the study. There were 27 open-ended questions to study demographic details of the patients and water, sanitation, and hygiene practices for under-five children in the study population. This was translated into the local language which was then back translated to English to check the validity of translation. All interviews were conducted in the local language by the first author (VB), who is fluent in Telugu. Each interview took about 30–40 minutes.

The institute's ethics committee approved the study. Necessary permissions were taken from the Chittoor district administration for conducting the study. Data were entered into a Microsoft Excel spreadsheet and analyzed with SPSS version 17.0 (Chicago, IL, USA). A summary WaSH score was generated as shown in Table 1. Wherever applicable, proportions and mean (SD) were calculated. *P* value of <0.05 was considered significant. The hierarchical stepwise multiple linear regression analysis was utilized to identify key demographic, socioeconomic, and housing determinants of WaSH score.

## 3. Results

A total of 500 children were studied. The demographic and socioeconomic characteristics of the study population are shown in Table 2. Majority of the households (69%) reported doing nothing at home to make the water safe for drinking. Over 93.8% of the households reported storing water in a utensil covered with a lid. Nearly three-quarters (74.2%) of the households reported cleaning the utensils at least once a day. Nearly 90% of the households reported retrieving water by dipping glass in the vessels, which were generally cleaned daily and covered (Table 3). Open defecation was commonly reported (84.8%) among the study population and mainly open drains and the street were the places of defecation for their children. Latrine use was only 4.0% among the households. Around 49.9% of mothers reported leaving stools of their under-three children uncovered (Table 3).

Less than two-thirds (59.4%) of the study households reported using water and soap for cleaning dirty hands. Merely one-third (37.4%) of the household members of the study participants reported using water and soap after defecation. Over 90% of the household members of the study participants reported cleaning their hands with water only before and after meals. Soap was used when hands were thought to be dirty. The hand wash score was more than 9 in 28.8% of the study participants. Nearly 90% of children reported bathing and changing clothes daily. In fact, about half of them reported doing this twice a day. Around 57% of the mothers reported cutting nails every 10 days. Most of the children were reported to brush their teeth at least once a day, usually with a toothbrush and paste. About 91% of children had the habit of eating raw vegetables and nearly 43.8% of them used to wash them with water before eating (Table 3).

**Figure 1:**
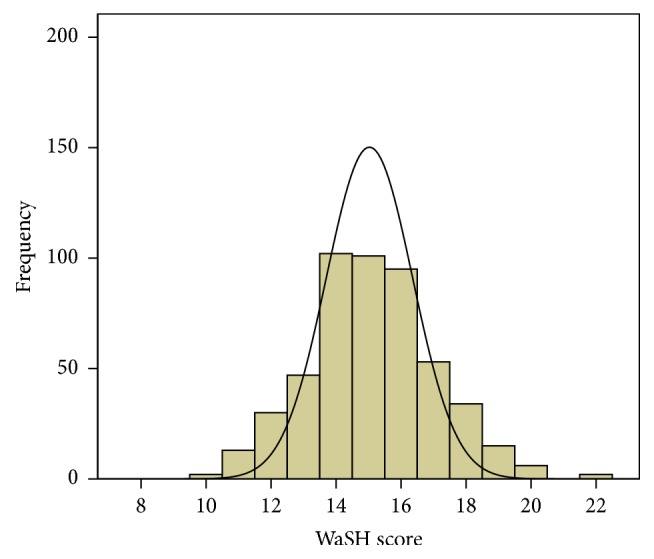
Distribution of households with respect to WaSH score.

The first quartile of WaSH score was 14, and the third quartile was 16. The median WaSH score was 15 (Figure 1). In the hierarchical stepwise multiple linear regression analysis, child demographic factors (Block 1, Table 4) explained 0.3% of the variance (adjusted *R*^2^ = 0.003) in total WaSH score when none of the other factors were controlled for. Socioeconomic variables (Block 2, Table 4) explained an additional nearly 14.5%. Altogether, the final model explained 14.3% of the variance in WaSH score. Mother's occupation and father's education and occupation were significantly associated with WaSH score in the hierarchical stepwise multiple linear regression analysis (Table 4).

## 4. Discussion

The most important way to reduce the spread of infections among children is clean water, basic toilets, and good hygiene practices. In the present study, the major source of drinking water among the study population was public taps. This was lower compared to DLHS-3. District Level Household Survey-3 (DLHS-3) in 2007-08 reported that, in Chittoor district, 99.6% of the households had a supply of drinking water at home [13]. Households with an improved drinking water source in Andhra Pradesh were 72.7% as reported by the National Family Health Survey-4 (NFHS-4) in 2015-16 [14]. Though nearly three-fourth of the study households cleaned the water storing utensils daily, majority of the households did not do anything for making water safe to drink. Moreover, more than 90% of the households practiced retrieval of water using a glass without a ladle. A study conducted in rural Chennai reported that around 45% of the participants were not using any methods of water treatment [15]. A community-based cross-sectional study in rural Kerala among 103 mothers of under-five children found that nearly 96.1% of the mothers used boiled water for drinking [16]. Without the basic water storage and retrieval practices, the lives of children of Sugali Tribes are at risk of water and sanitation related diseases leading to mortality.

Majority (84.8%) of the household members of the study children were practicing open field defecation and 83.6% of their under-five children were also practicing the same, which increased the risk of waterborne diseases. Around half of the mothers used to leave stools of their under-three children uncovered. The situation among the tribal population seems to be worse as compared to the whole of Chittoor district as per the DLHS-3 findings that 33.3% of the households in Chittoor district, Andhra Pradesh, had toilet facilities [13]. The practice of open defecation was present in 78% of ST households against 48% of all households in Andhra Pradesh. In Andhra Pradesh, 80.5% of ST households did not have a sanitary latrine facility within the premises [2]. The proportion of households practicing open field defecation was very high compared to a study rural area of Chennai, which stated that around one-fourth of the study participants use community toilets, open defecation, or sharing of toilets [15]. This shows the recent integration of tribal communities into modern society.

Less than two-thirds of the household members of the study used water and soap for cleaning dirty hands. Over 90% of the household members of the study participants cleaned their hands only with water before and after meals. Merely one-third of the household members of the study participants were using water and soap after defecation. This was higher than that reported by HUNGaMA survey, where 10.8% used soap to wash hands before meals and 19% washed hands after using the toilet [17]. A study from urban slums of West Bengal and Tripura reported that over 90% of the study population practiced hand washing after defecation [18]. Our finding was lower compared to a study conducted among 57 mothers in Indonesia which reported that 43% of the mothers use soap to wash their and their child's hands after defecation [19]. The scarcity of water influences hand washing. Gaps in hygiene practices of Sugali Tribe's households are a matter of public health concern.

The mother's and father's education levels were associated with significantly higher WaSH score, indicating better WaSH practices in the present study. Likely, a community-based cross-sectional study conducted in a rural area of Kerala among 103 mothers of under-five children found better water and hygiene practices due to higher education status of mothers [16]. A cross-sectional study conducted in slums of Hyderabad, Andhra Pradesh, among 506 households, of 451 children aged 6–59 months, stated that improved knowledge of caregivers was associated with higher odds of better child hygienic practices [20]. Similarly, a health facility based cross-sectional study conducted in Port Harcourt among 154 mothers of under-five children found that self-reported hand washing with soap and water before feeding the child and after cleaning the child was significantly associated with higher mothers' level of education [21]. This might be primarily due to changes in traditional beliefs, attitude, and improved WaSH practices in them. Working and nonlaborer fathers had better WaSH practices. This could be mainly due to better child health care seeking decisions.

The population has better practices in certain areas of WaSH like water storage, waste disposal, and child body hygiene, while not so high in the other. This is because while WaSH is clubbed as one domain, it subsumes multiple domains like access to water supply and sanitation infrastructure, social customs and habits, and so forth. Poor WaSH mainly included not washing hands with soap after defecation and before and after eating food, eating unwashed fruits and vegetables, open field defecation, and eating raw vegetables. Predisposing factors for poor WaSH were water scarcity, especially in summers, and lack of access to drinking water. There are some limitations of this study. Study of WaSH practices was based on a report by the informant and some degree of recall bias cannot be ruled out [22]. For a better understanding of behavior, which especially focuses on WaSH practices of a tribal population, one needs a qualitative study, which was beyond the scope of this study.

## 5. Conclusion

Water, sanitation, and hygiene practices are one of the largest causes of morbidity and mortality in children. The present study found a need for improvement in WaSH practices of the people of Sugali Tribes, especially those related to the use of sanitary latrines, hand washing, and water treatment practices. Better integration into the society with a subsequent increase in access to sanitation infrastructure, economic schemes, and educational interventions is necessary for further improvements. A community-based intervention program needs to be carried out to educate the tribal people about appropriate water storage and retrieval methods and sanitation and hand washing practices.

## Figures and Tables

**Table 1:** Indicators of water, sanitation, and hygiene practices that were used to develop summary WaSH score.

Drinking water score^*∗∗*^	Sanitation score^*∗∗*^	Hygiene score^*∗∗*^	Hand wash score^*∗∗*^
*Water supply*	*Defecation practice*	*Child body hygiene*	When hands are dirty^*∗*^ After defecation^*∗*^ Before preparing food^*∗*^ Before eating food^*∗*^ After eating food^*∗*^ After cleaning children^*∗*^
Piped water supplied to the house Distance of water supply less than 100 meters	Use of sanitary latrine in the house by household members Use of sanitary latrine in the house by children	Daily bathing Daily changing of clothes Daily brushing of teeth	
*Water storage*	*Waste disposal*	*Food hygiene*	
Daily cleaning of vessels Covering utensils with a lid Treating water to make it safe	Waste pit away from the house or collected by municipal person	Washing before eating fruits and vegetables Do not eat raw vegetables	
*Water retrieval*			
Tap connected to vessel/tank or drawn by ladle/vessel with a handle			

Possible score 0–6	Possible score 0–3	Possible score 0–5	Possible score 0–12

^*∗*^Score of various hand washing methods: wash with water and soap = 2, wash with only water = 1, and do not wash = 0.

^*∗∗*^WaSH score. It includes 4 broad indices: drinking water score, sanitation score, hygiene score, and hand washing score. A summary WaSH score was generated from the sum of the four specific indices and had a total of 26 points possible.

**Table 2:** Demographic and socioeconomic characteristics of the study population (*n* = 500).

Variable	Category	Total *n* (%)
Child characteristics		
Age of the child in months	0–11	124 (24.8)
	12–35	228 (45.6)
	48–59	148 (29.6)
Gender of the child	Girls	244 (48.8)
	Boys	256 (51.2)
Birth order	1	343 (68.6)
	>1	157 (31.4)
Socioeconomic characteristics		
Mother's education	≥ primary school	240 (48.0)
	< primary school	260 (52.0)
Father's education	≥ primary school	165 (33.0)
	< primary school	221 (44.2)
Mother's occupation	Nonlaborer	221 (44.2)
	Laborer and not working	279 (55.8)
Fathers' occupation	Nonlaborer	148 (29.6)
	Laborer and not working	352 (70.4)
Household characteristics		
Type of house	Pucca	453 (90.6)
	Semipucca/kutcha	47 (9.4)
Presence of overcrowding^*∗*^	Present	326 (65.2)
	Absent	174 (34.8)
Below poverty line status^*∗∗*^		196 (39.2)

^*^Overcrowding is defined using the number of persons per room criteria.

^**∗∗**^BPL criteria used annual family income up to Rs 60,000.

**Table 3:** Reported water (W), sanitation (S), and hygiene (H) practices among household members (*n* = 500).

Drinking water (*N* (%))	Sanitation (*N* (%))	Hygiene (*N* (%))	Hand washing		
			*Occasion*	*With soap and water (N (%))*	*Only water (N (%))*
*Water supply*	*Defecation practice*	*Child body hygiene*	When hands are dirty	297 (59.4)	186 (37.2)
Presence of piped water in the house (10 (2.0))	Use of sanitary latrine in the house for defecation by household members (73 (14.6))	Daily bathing (444 (88.8))	After defecation	187 (37.4)	263 (52.6)
Distance of water supply less than 100 meters (461 (92.2))	Use of sanitary latrine in the house for defecation by children (65 (13.0))	Daily changing of clothes (444 (88.8))	Before preparing food	46 (9.2)	417 (83.4)
*Water storage*	*Waste disposal*	Daily brushing of teeth (356 (71.2))	Before eating food	38 (7.6)	460 (92.0)
Frequency of cleaning of vessels daily (371 (74.2))	Waste pit away from the house/collected by municipal person (416 (83.2))	*Food hygiene*	After eating food	5 (1.0)	493 (98.6)
Covering utensils with lid (469 (93.8))		Washing before eating fruits and vegetables (219 (43.8))	After cleaning children	166 (33.2)	286 (57.2)
Do some procedures for making water safe (155 (31.0))		Do not eat raw vegetables (95 (19.0))			
*Water retrieval*					
Tap connected to vessel/tank & drawn by vessel with a handle (45 (9.0))					

**Table 4:** Results of hierarchical stepwise multiple linear regression analyses.

Analysis block	Adjusted *R*-square	Independent variable	*B*	95% CI of *B*		*β*	*P* value
				Lower bound	Upper bound		
Dependent variable = WaSH score							

*Block 1*							
Demographic factors	0.003	Child gender	–0.091	–0.416	0.234	–0.023	0.583
		Child age	–0.154	–0.394	0.085	–0.058	0.206
		Child birth order	–0.212	–0.935	0.511	–0.050	0.565

*Block 2*							
Socioeconomic factors	0.145	Mother's education	–0.209	–0.575	0.156	–0.054	0.261
		Mother's occupation	–0.679	–1.063	–0.295	–0.172	0.001
		Father's education	–0.426	–0.793	–0.058	–0.102	0.023
		Father's occupation	–0.803	–1.212	–0.395	–0.188	0.000
		BPL family	0.195	–0.153	0.544	0.049	0.270

*Block 3*							
Housing factors	0.143	Pucca house	–0.214	–0.769	0.342	–0.032	0.450
		No Overcrowding	–0.070	–0.760	0.621	–0.017	0.843

*B* is the unstandardized regression coefficient.
